# A Comparative Study of Polysomnography‐Derived Sleep Disturbance in People Living With Multiple Sclerosis Compared to Matched Controls From the General Population

**DOI:** 10.1111/jsr.70075

**Published:** 2025-04-28

**Authors:** Amy C. Reynolds, Emma Thomas, Yohannes Adama Melaku, Hanna A. Hensen, Arun V. Krishnan, Simon C. Gandevia, Stephen R. Lord, Peter R. Eastwood, Danny J. Eckert

**Affiliations:** ^1^ Flinders Health and Medical Research Institute (Sleep Health) Flinders University Bedford Park South Australia Australia; ^2^ Neuroscience Research Australia (NeuRA) Sydney New South Wales Australia; ^3^ School of Medical Sciences University of New South Wales Sydney New South Wales Australia; ^4^ School of Clinical Medicine, Faculty of Medicine and Health, UNSW Sydney New South Wales Australia; ^5^ Department of Neurology Prince of Wales Hospital Sydney New South Wales Australia; ^6^ Prince of Wales Clinical School, University of New South Wales Sydney New South Wales Australia; ^7^ Health Futures Institute Murdoch University Murdoch Western Australia Australia

**Keywords:** chronic disease, multiple sclerosis, obstructive sleep apnea, periodic limb movement disorder, polysomnography, sleep wake disorders

## Abstract

Sleep structure and sleep disorders were compared between people with multiple sclerosis (MS; *n* = 39) and age, sex, and BMI‐matched members of the general population (*n* = 39) using overnight polysomnography (PSG). Compared to population controls, people with MS had a higher prevalence of periodic limb movement disorder (PLMD; 59% vs. 18%, *p* < 0.001) and PLM‐related arousals (PLMI: 21.1 vs. 0.8, *p* < 0.001); as well as longer sleep duration (402.9 [59.8] vs. 370.4 [54.0] min, *p* = 0.014), longer median sleep latency (12.4 min) and a reduced proportion of total sleep time in stage N1 sleep (8.5% vs. 14.8%, *p* < 0.001) and more time in N2 sleep (54.4% vs. 48.0%, *p* < 0.001). Sleep architecture appeared to differ for people with MS, even in the context of no recent exacerbations or relapse. Managing periodic leg movements during sleep may help improve sleep quality in people with MS.

## 1 Introduction

People living with multiple sclerosis commonly report fragmented, disturbed and poor quality sleep (Zhang et al. 2023). These sleep problems are associated with reduced well‐being, as well as increased disease activity and fatigue (Hensen et al. 2017). Early identification and treatment of sleep‐related risk factors may help improve quality of life and well‐being in people with MS.

Identification of ways to treat sleep problems in people with MS is challenging due to lack of clarity in how MS affects sleep and its associated disorders. This is due, in part, to limitations of existing studies on sleep in people with MS including: a reliance on potentially biased study populations referred specifically due to reported sleep problems, or confounded by active disease flares; limited use of gold‐standard sleep measurement (overnight polysomnography) to quantify sleep (Brass et al. 2014; Nociti et al. 2017); and infrequent use of population controls without MS when interpreting sleep data. Thus, this study used polysomnography to characterise sleep structure and sleep disorders in people with MS who had experienced no MS exacerbations or relapses for over 30 days. Their results were compared to matched control participants from the general population.

## 2 Methods

### 2.1 Participants

#### 2.1.1 Multiple Sclerosis (MS)

Fifty‐four participants were recruited consecutively from a larger randomised trial cohort of people with MS undergoing an exercise intervention with a falls‐related primary endpoint (Hoang et al. 2024). The participants for this analysis were asked to participate in a home‐based sleep sub‐study involving Level 2 polysomnography prior to commencement of the exercise intervention.

#### 2.1.2 Matched Controls

A community sample of West Australian adults with Level 1 (laboratory‐based) sleep studies was matched to people with MS with respect to age, sex and body mass index (BMI). These participants were a convenience sample of the parents (Generation 1, Gen 1; *n* = 1098) of participants (Generation 2, Gen 2) in the longitudinal Raine Study (ethics approval Human Research Ethics Committee, University of Western Australia, RA/4/1/7236). A detailed analysis of sleep in this population was reported previously (McArdle et al. 2022).

### 2.2 Polysomnography‐Based Sleep Measurements

People with MS completed an overnight sleep study in their home (Nox A1, Noxturnal, Reykjavik, Iceland). Data from community controls were collected from an overnight in‐laboratory sleep study (Grael, Compumedics, Abbostford, Vic., Australia) conducted at the Centre for Sleep Science, University of Western Australia. A minimum of 4 h of sleep recording was required for inclusion in the present analysis, and sleep staging and signal analyses were performed in accordance with the American Academy of Sleep Medicine (AASM) guidelines by experienced scorers, who were blinded to other sleep‐ or disease‐related data (McArdle et al. 2022) (see Table 1).

**TABLE 1:** Sample characteristics, sleep architecture and sleep disorders for people with multiple sclerosis and population controls.

	Multiple sclerosis		Population controls		*p*
	Median (IQR)	Range	Median (IQR)	Range	
Sample characteristics					
Age (years)^a^	55.1 (9.3)	39–72	55.3 (9.0)	40–70	0.900
Sex (female, *n* [%])	30 (77.0)		30 (77.0)		1.000
BMI	25.4 (5.4)	20.0–41.9	26.3 (5.1)	21.5–41.6	0.591
PHQ‐9	8.0 (9.0)	0.0–23.0	3.0 (5.0)	0.0–21.0	0.006
Diabetes, *n* (%)	5 (12.8)		4 (10.5)		0.754
Hypertension, *n* (%)	9 (23.1)		10 (25.6)		0.792
Heart disease, *n* (%)	4 (10.3)		2 (5.4)		0.433
Self‐reported sleep variables					
ESS total score	7.0 (6.75)	0.0–20.0	7.0 (6.0)	1.0–15.0	0.098
PSQI total score	7.0 (6.0)	1.0–18.0	5.5 (5.0)	1.0–17.0	0.055
PSG‐derived sleep variables					
Sleep efficiency (%)	82.2 (11.8)	50.6–95.0	83.9 (11.6)	52.5–97.8	0.773
Wake after sleep onset (min)	58.0 (38.2)	14.0–220.0	57.0 (12.2)	10.5–139.0	0.734
N3 (%TST)^a^	13.2 (7.3)	0.0–25.9	14.3 (6.5)	0.3–29.0	0.489
REM (%TST)^a^	21.9 (7.4)	0.0–39.8	20.8 (6.0)	7.3–34.4	0.460
Sleep disorders, *n* (%)					
Insomnia	11 (28.2)		8 (20.5)		0.429
OSA (AHI ≥ 15 events/h)	8 (20.5)		9 (23.1)		0.784
RLS	10 (25.6)		4 (10.3)		0.068
PLMD	23 (58.9)		7 (17.9)		< 0.001

*Note*: Variables with significant case–control differences (total sleep time, time in bed, sleep onset latency, N1% and N2% and apnea–hypopnea index are reflected visually in Figure 1).

Abbreviations: AHI, apnoea–hypopnea index; ESS, Epworth Sleepiness Scale; OSA, obstructive sleep apnea; PLM, periodic limb movement; PLMD, periodic limb movement disorder; PLMI, periodic leg movement index; PSG, polysomnography; PSQI, Pittsburgh Sleep Quality Index; RLS, restless legs syndrome; TST, total sleep time.

^**a**^ Data reported as mean (standard deviation, SD); all other values are reported as median (interquartile range, IQR) or *n* (%).

### 2.3 Questionnaire‐Based Sleep Measurements

Validated sleep questionnaires were used in both groups (Pittsburgh Sleep Quality Index [PSQI] and the Epworth Sleepiness Scale [ESS]). Insomnia in people with MS was determined using the Insomnia Severity Index (ISI). Chronic insomnia in the controls was determined from the Pittsburgh Sleep Symptom Questionnaire‐Insomnia (PSSQ‐I), with an additional duration criterion of ≥ 3 months instead of ≥ 4 weeks to align with current diagnostic criteria (McArdle et al. 2022). Restless legs syndrome was established with the 2003 International Restless Legs Syndrome Study Group (IRLSSG) diagnostic criteria for both MS and matched controls (Allen et al. 2014).

### 2.4 Statistical Analysis

Data were analysed using R (R Core Team, 2021, Vienna, Austria). Continuous variables are presented as either median (interquartile range, Med [IQR]) or mean (standard deviation, Mean [SD]) according to distribution. Categorical data are presented as numbers and percentages. Between‐group comparisons of people with MS and controls were made using Pearson's chi‐squared test for categorical variables, and either independent samples *t*‐test or Wilcoxon rank‐sum test for continuous variables. A two‐tailed *p* value of < 0.05 was considered statistically significant.

## 3 Results

### 3.1 Participants

**FIGURE 1:**
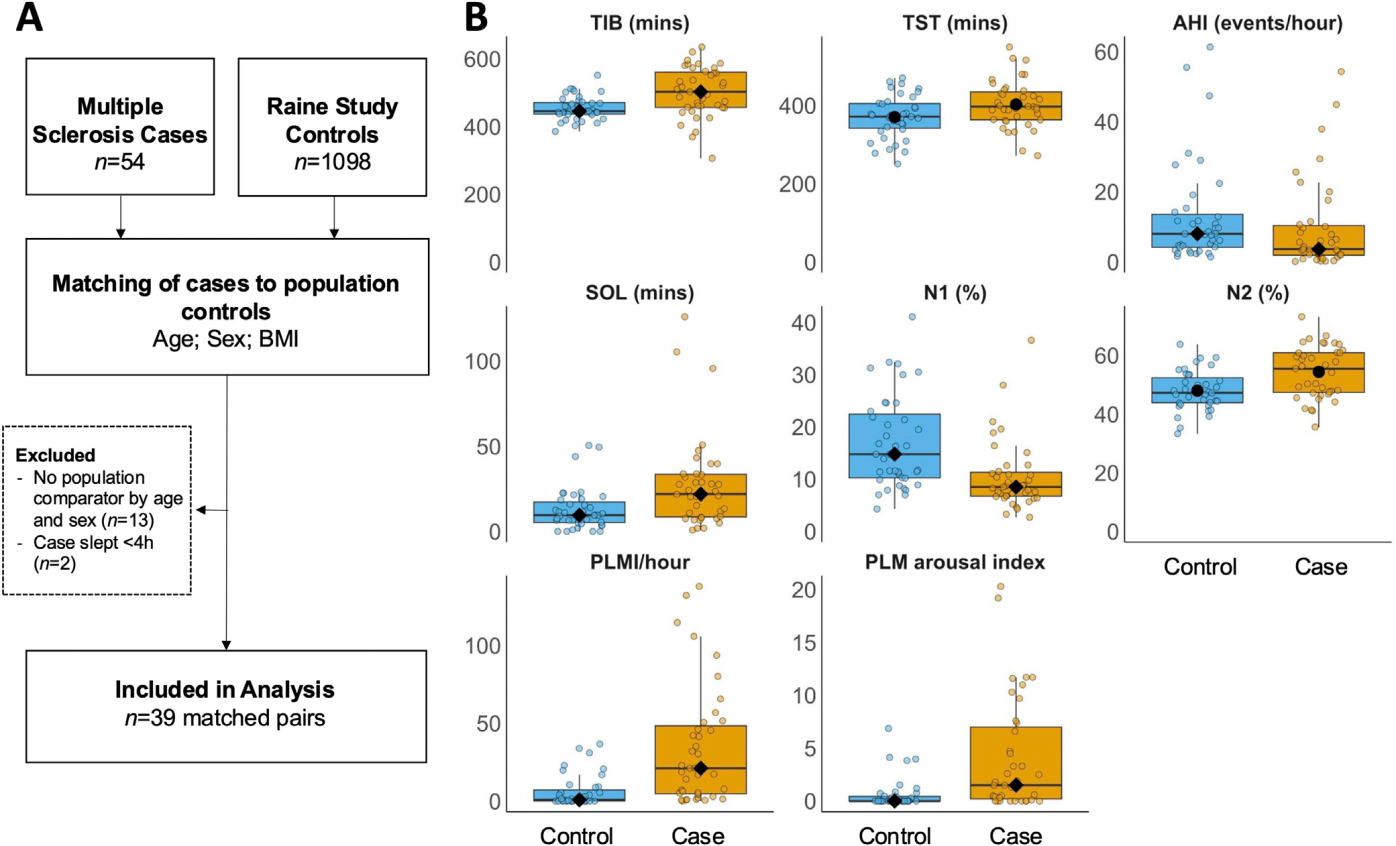
(A) Study flow diagram showing the process of selecting and matching people with MS with population controls, and (B) Polysomnographic sleep variables, showing statistically significant case–control differences. Normally distributed data are reflected as (●); non‐normally distributed data are reflected as (♦). AHI, apnea hypopnea index; AI, arousal index; N1–N3, sleep stages; PLMI, periodic leg movement index; REM, rapid eye movement sleep; SE, sleep efficiency; SOL, sleep onset latency; TIB, time in bed; TST, total sleep time; WASO, wake after sleep onset.

The process of selecting and matching cases and controls is shown in Figure 1. MS cases were closely matched on age, sex and BMI (Table 1). Prevalence of diabetes, hypertension and heart disease were similar between people with MS and control participants, while median scores reflecting depressive symptoms according to the Patient Health Questionnaire‐9 (PHQ‐9) were higher in people with MS than the controls. In those with MS, median disease duration was 13.0 (12.0) years. The mean Expanded Disability Status Scale (EDSS) score was 4.2 (1.2), and the mean Modified Fatigue Impact Scale (MFIS) score was 40.9 (16.3). Additionally, 38% reported using psychotropic medications, while 15% reported benzodiazepine use. No study participants were using continuous positive airway pressure (CPAP) therapy during overnight studies.

### 3.2 Sleep Characteristics

Sleep variables which differed significantly between the groups are reported in Figure 1B. Self‐reported sleepiness was not significantly different between people with MS and controls. Poor sleep quality, reflected by higher median PSQI scores, was apparent in people with MS, although both groups reported median PSQI values which exceeded the cut point for poor sleep quality (≥ 5, Table 1; *p* = 0.055). Median [IQR] study duration (MS vs. control = 502.4 [104.3] vs. 445.5 [34.6] min) and mean total sleep time were longer for people with MS than controls (402.9 [59.8] min, or 6.7 h, vs. 370.4 [54.0] min, or 6.1 h, *p* = 0.014). Sleep onset latency (SOL) was significantly longer in people with MS compared to controls (Med = 21.9 [25.0] vs. 9.5 [12.0], *p* < 0.001), while sleep efficiency (SE) and wake after sleep onset (WASO) were not different between the groups.

Sleep architecture differed, with a lower median percentage of time in Stage N1 (MS vs. control = 8.5 [4.5] vs. 14.8 [12.2], *p* < 0.001) and a higher average percentage time in Stage N2 (MS vs. control = 54.4 [8.8] vs. 48.0 [6.7], *p* < 0.001) in people with MS. Stage N3 and Stage REM as a percentage of total sleep time were not different between the groups.

The apnoea–hypopnea index was within the normal range in both groups, although marginally higher in the control group (8.0 [9.4]) compared with people with MS (3.6 [8.5]; *p* = 0.032). Conversely, people with MS had significantly higher median periodic limb movement index (PLMI; 21.1 [43.7] vs. 0.8 [7.2], *p* < 0.001) and periodic limb movement (PLM) arousal index (1.5 [6.8] vs. 0.0 [0.4], *p* < 0.001) than controls. Within MS patients, PLMI, PLM arousal index and PLMD prevalence were not significantly different between those with and without reported psychotropic medication use (*p* = 0.62, 0.26 and 0.44, respectively).

### 3.3 Sleep Disorders

A significantly higher rate of periodic limb movement disorder (PLMD) was observed in people with MS compared to controls, as indicated by PLMI > 15 events/h (Walters et al. 2007). There were no significant differences in other clinical sleep disorders between people with MS and control participants (Table 1).

## 4 Discussion

The main findings of this study are that compared with age, sex and BMI‐matched population controls, people with MS with stable disease have an increased prevalence of PLMD at nearly 60%. Other common clinical sleep disorders occurred at comparable rates between groups. Sleep disorder prevalence was high, with 77% of MS participants and nearly 50% of age, sex and BMI‐matched population controls meeting criteria for at least one common sleep disorder.

Higher PLM values in people with MS are consistent with recent findings (Ferri et al. 2022), and may relate to the presence of lesions in descending motor pathways and associated impaired muscle function. It is possible that such lesions may not be mirrored in corresponding sensory pathways, which aligns with our observation of increased limb movements despite the typical discomfort characteristic of restless leg syndrome (RLS). In contrast to previous findings (Brass et al. 2014), median rates of self‐reported RLS in people with MS did not differ from population controls (*p* = 0.07). Rates of moderate to severe sleep apnoea were comparable between people with and without MS (Braley et al. 2012) and consistent with overall global prevalence estimates of sleep apnoea (Lechat et al. 2023).

AHI differences between our findings and the work of Braley and colleagues may be attributable, at least in part, to the absence vs. presence of MS disease activity at the time of measurement, which could disrupt sleep and perpetuate sleep disordered breathing. The use of home sleep studies in people with MS may have also underestimated sleep apnoea severity (Ghegan et al. 2006). Use of home sleep studies may have also contributed to differences in SOL between groups. However, this is unlikely, as people with MS in the current study had similar mean SOL (21.9 min) to SOL observed in other studies (20.3 min) (Zhang et al. 2023). Future longitudinal investigations and in‐laboratory polysomnography in people with MS during periods of relapse and remitting disease would be insightful to better understand the relationship between disease activity and sleep disturbance, and address potential impacts of Level 1 vs. Level 2 studies. Use of non‐invasive wearable and nearable sleep monitoring approaches may also be a cost‐effective approach to investigate this objective over multiple nights in the home environment (Lechat et al. 2023; Pinilla et al. 2025).

In most previously published studies, MS and control groups have been drawn from patients referred for sleep studies, rather than community‐based samples which could bias the findings and interpretation (Zhang et al. 2023), and potentially explain our differing findings related to OSA. For example, some previous studies have selected controls who presented to sleep clinics, which likely do not reflect the general population (Dubessy et al. 2021). Others have drawn on community cohorts as controls, but studied participants with more heterogeneous MS disease severity, and did not report on insomnia or PLMD (Sparasci et al. 2022). While the present study found lower N1% and higher N2% in people with MS compared to community controls, N3% and REM% were similar. It is plausible that this could be related, at least in part, to home vs. in‐laboratory monitoring (Iber et al. 2004). This requires confirmation with comparable polysomnography approaches (Level 1 or 2) for both groups in future studies.

Given the potential relationship between increased sleep problems and relapse, targeting sleep problems may provide an ideal opportunity for clinical intervention. Indeed, sleep disturbance has been identified as a risk factor for MS relapse (Sahraian et al. 2017). Sleep disorders treatment may be an important target to improve outcomes for people with MS (Hensen et al. 2017), and those in the general population broadly.

A key strength of the present study is the use of community control participants who were closely matched with the MS participants based on age, sex and BMI. Further, both groups completed polysomnography. This enables accurate comparison of sleep parameters between groups using gold‐standard equipment including electroencephalogram, electrooculogram to quantify sleep, leg movements using electromyography and respiratory parameters based on flow (nasal pressure) and oxygen saturation.

Our findings should be considered in the context of some limitations. People with MS completed sleep studies in their home with more flexibility for bed and wake time, compared to Level 1 studies in control participants which were in‐laboratory and confined to a prescribed sleep window opportunity. This may explain the differences in sleep time and SOL observed, although these differences will have less effect on measures reported as indices, such as PLMI, which were markedly increased in participants with MS relative to controls. Further, while MS patients did not differ on PLM arousal index and PLMI according to psychotropic medication use in our sample, it would also be important in future studies to consider the potential impact during periods of higher disease severity as some medications for MS can influence sleep architecture (Brass et al. 2014). The origin of the elevated PLMI in people with MS in the current study is unclear. However, it is possible that nocturnal spasticity as a consequence of MS, rather than classic dopaminergic dysfunction, may have played a contributing role. This requires further investigation as it may have important therapeutic implications on how best to manage the high rates of PLMs in people with MS.

## 5 Conclusion

In conclusion, even in the absence of recent relapsing disease activity, people with MS report high rates of at least one sleep disorder, predominantly related to PLMD. Considering any changes with relapsing disease activity will be important, particularly given that PLMD rates are higher in people with MS even in the context of low disease activity. Relapsing disease may yield even higher rates of PLMD and other sleep disorders. Sleep disorder screening and treatment may represent an important target for care in people with MS, and managing PLMD may support improved sleep.

## Author Contributions

**Amy C. Reynolds:** writing – original draft, writing – review and editing, conceptualization, visualization, project administration, formal analysis, data curation. **Emma Thomas:** writing – review and editing. **Yohannes Adama Melaku:** formal analysis, writing – original draft, writing – review and editing, methodology. **Hanna A. Hensen:** conceptualization, investigation, writing – review and editing, data curation. **Arun V. Krishnan:** data curation, conceptualization, investigation, writing – review and editing. **Simon C. Gandevia:** conceptualization, investigation, writing – review and editing, data curation. **Stephen R. Lord:** conceptualization, investigation, data curation, writing – review and editing. **Peter R. Eastwood:** methodology, writing – review and editing, project administration, data curation. **Danny J. Eckert:** conceptualization, funding acquisition, writing – original draft, writing – review and editing, validation.

## Ethics Statement

This study recruited people with MS enrolled in a falls‐related interventional study (ACTRN12616001053415) approved by the South Eastern Sydney Local Health District Human Research Ethics Committee (14/312). Matched controls form a sub‐population of the longitudinal Raine Study, which has been approved by the Human Research Ethics Committee, University of Western Australia (approval number: RA/4/1/7236). The overarching analysis for this study was approved by Flinders University Human Research Ethics Committee (HEL4716‐1, 2021).

## Conflicts of Interest

Danny J. Eckert, Simon C. Gandevia, Stephen R. Lord and Yohannes Adama Melaku are supported by National Health and Medical Research Council of Australia Leadership Fellowships. Amy C. Reynolds is supported by an Australian Research Council Discovery Early Career Researcher Award. No further conflicts of interest are reported related to this analysis.

## Data Availability

Data may be available upon request by email to MS study senior investigators (Prof Danny Eckert, danny.eckert@flinders.edu.au) subject to ethical approval. Community controls data were provided through data request and approval by the Raine Study. Contacts for data access related to these data should be directed to rainestudy@uwa.edu.au.
